# Optical analogues of the Newton–Schrödinger equation and boson star evolution

**DOI:** 10.1038/ncomms13492

**Published:** 2016-11-14

**Authors:** Thomas Roger, Calum Maitland, Kali Wilson, Niclas Westerberg, David Vocke, Ewan M. Wright, Daniele Faccio

**Affiliations:** 1 School of Engineering and Physical Sciences, Heriot-Watt University, Edinburgh EH14 4AS, UK; 2 College of Optical Sciences, University of Arizona, Tucson, Arizona 85721, USA

## Abstract

Many gravitational phenomena that lie at the core of our understanding of the Universe have not yet been directly observed. An example in this sense is the boson star that has been proposed as an alternative to some compact objects currently interpreted as being black holes. In the weak field limit, these stars are governed by the Newton–Schrodinger equation. Here we present an optical system that, under appropriate conditions, identically reproduces such equation in two dimensions. A rotating boson star is experimentally and numerically modelled by an optical beam propagating through a medium with a positive thermal nonlinearity and is shown to oscillate in time while also stable up to relatively high densities. For higher densities, instabilities lead to an apparent breakup of the star, yet coherence across the whole structure is maintained. These results show that optical analogues can be used to shed new light on inaccessible gravitational objects.

## Abstract (web summary)

In the weak field limit, boson star evolution is governed by the Newton-Schrödinger equation. Here the authors report an optical setup that provides a formal analogue of such dynamics via the interaction between vortex beams and a medium with positive thermo-optical nonlinearity.

Analogue gravity is the study of gravitational effects using artificial systems that recreate some specific aspects of the full gravitational system using laboratory-based experiments^1^. Recent experimental studies have focused attention on analogues for black holes and the search for Hawking radiation in transonic fluid flows^2^ that may be obtained in a variety of systems such as flowing water^3^, Bose–Einstein condensates^4^,^5^ and nonlinear optics^6^,^7^,^8^. To date, attempts to experimentally study gravitational systems using analogues have tended to focus on the local and linear dynamics, that is, on the behaviour of weak perturbations or waves in background curved spacetime geometry, determined solely by the local flow speed of the supporting fluid or medium. However, gravity is inherently a nonlinear and nonlocal, that is, long-range, interaction. This has been used to draw an analogy between gravitational attraction and light-trapping in the wake of optical solitons^9^. Another notable example of the connection between gravity and optics was recently demonstrated using an optical system based on a thermally excited medium, which allows one to reproduce the physics of the Newton–Schrödinger equation (NSE)^10^. The NSE can be written as


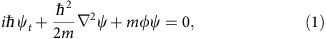


which describes the evolution of a particle with mass *m* defined by a wave function 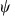. ∇^2^ is the three-dimensional (3D) Laplacian and the subscript *t* denotes a derivative with respect to time. The gravitational potential *φ* is determined by the Poisson equation


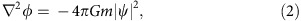


where *G* is the gravitational constant. This equation was proposed by Diòsi^11^ and Penrose^12^ as an attempt to investigate quantum wave function collapse in the presence of a Newtonian gravitational potential. More recently, the same equation has been used, as in its original proposal^13^ to describe the evolution of boson or Bose–Einstein condensate stars. Indeed, the NSE may be obtained as the non-relativistic limit of the Klein–Gordon equation^14^, and describes the coupling of classical gravitational fields to quantum matter states. Importantly, and in departure from most previous ‘linear' analogue gravity studies, here the gravitational potential includes the quantum matter itself as a source. The remarkable feature that allows one to build laboratory experiments is that the evolution of the amplitude 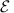 of an optical beam in a thermally focusing medium is described by


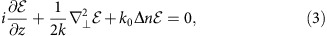


where 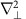 is the transverse, two-dimensional (2D) Laplacian, *k*=*n*_b_*ω*/*c*=*n*_b_*k*_0_, *n*_b_ being the background refractive index and the nonlocal change in refractive index, Δ*n* is induced by heating of the medium by the beam itself


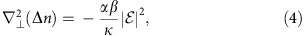


where *β*=∂*n*/∂*T* is the medium thermo-optic coefficient, *κ* is the thermal conductivity and *α* the absorption coefficient.

The similarity between the gravitational equations (1) and (2) and the nonlinear optical beam propagation equations (3) and (4) indicates the possibility to simulate a 2D slice of the full 3D gravitational system. We note that in the optical model, the propagation of the optical beam through space (*z*) maps onto the time coordinate of the NSE.

In this article we show that if care is taken in choosing the appropriate operating conditions, the medium response in the transverse plane of the optical beam can be made to mimic exactly the gravitational scenario. More precisely, we find that exact correspondence is obtained when the optical beam carries no energy on-axis in the Fourier domain, that is, when the pump is ring-shaped in K-space. We then use this correspondence to experimentally study the evolution of a rotating boson star that has quantized angular momentum^15^,^16^ using an intense light beam propagating in a lead-glass slab. For low star densities (beam intensities) the evolution is characterized by self-focusing contraction cycles. The experimental results are well reproduced by numerical simulations, which are then extended to higher densities than those achievable in the experiments. At these high densities, the star becomes unstable yet complete collapse is prevented by the phase singularity (related to the quantised angular momentum of the entire structure) at the star centre.

## Results

### Theoretical model

The basic model for an optical beam propagating in a thermo-optic medium is derived within the paraxial approximation and incorporates the thermally induced change in refractive index Δ*n*(**r**_⊥_, *z*), **r**_⊥_=(*x*, *y*) being the transverse position vector. The resulting paraxial wave equation for the slowly varying complex electric field envelope propagating along the *z* axis is


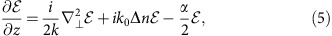


where 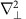 is the transverse Laplacian, *α* is the medium absorption coefficient, and the nonlocal change in refractive index is expressed by the integral^17^,^18^


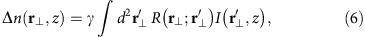


where *I*(**r**_⊥_, *z*)=|
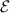
(**r**_⊥_, *z*)|^2^ describes the beam intensity profile and 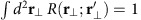. Thus, in the case of a local nonlinear optical response, 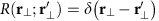, the nonlinear coefficient is given by *γ*. In the above equations, 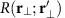 is the response function for the medium, which incorporates the transverse boundary conditions. We assume the transverse boundary does not vary with *z*, that the medium is sufficiently long, and optical absorption low enough that the thermal diffusion is dominated by transverse diffusion and longitudinal effects are not significant.

### Response function

It is by tailoring the medium response function that one may ultimately reproduce a precise analogue of the NSE in the laboratory. Physically the change in refractive index in the medium arises from the temperature increase due to laser absorption Δ*n*=*β*Δ*T*, with *β*=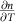. Taking into account appropriate boundary conditions, the spatial profile of the temperature change obeys the heat equation


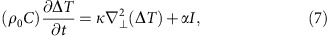


with *κ* the thermal conductivity and *ρ*_0_*C* the heat capacity per unit volume. Finally, in steady state, found by setting the time derivative to zero, we obtain equation (4) which should be solved subject to the appropriate boundary conditions due to heat loss. Equation (6) shows that the response function is, to within a constant, the Green's function for heat diffusion in the medium, and obeys


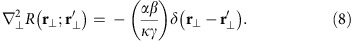


### Infinite space model

In this case the boundaries are moved off to infinity in the two transverse dimensions, and we might hope to have a realization of the Schrödinger–Newton equation albeit in 2D. Fourier transforming equation (8) yields


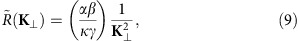


where the tilde signifies a transformed variable and 
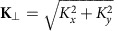
. The same functional form for 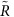 is of course also expected for the gravitational case. However, in the two dimensions used in our experiments (the transverse plane of our optical beam), the resulting response function in the real-space domain is


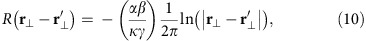


which is very different from the Coulomb-like (decaying) interactions that arises in three dimensions and in the gravitational case.

### Distributed loss model

The lesson from the infinite space model is that thermal losses due to the presence of the boundaries will always have to be incorporated at some level. We consider the case for which the boundaries are present but well removed by a characteristic distance *D*/2 from the transverse position at which the laser beam of width *W*<<*D*/2 is centred. For a cylindrical medium, *D* will be the medium diameter, whereas for a medium of rectangular cross-section, *D* is chosen as the smaller dimension. Then to a reasonable approximation the medium will be shift-invariant for displacements in the vicinity of the beam centre, that is, the boundaries should not significantly impact the symmetry of the laser beam as it propagates. In this regime we may include the effect of the distant boundaries into a distributed loss term in the starting temperature equation


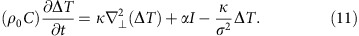


**Figure 1:**
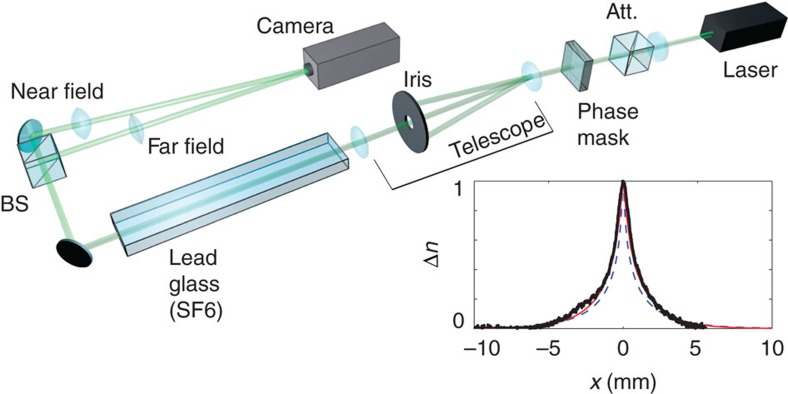
Experimental layout. The NSE is simulated using a continuous-wave laser centred at 532 nm, which is passed through an attenuator (Att., *λ*/2-waveplate+polarizer) then through an azimuthal phase mask. The phase mask imprints a vortex phase structure 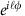 creating a series of diffracted orders. The first order (with 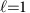) is isolated with an iris in the far field of an *f*=1 m lens and collected with an *f*=250 mm lens forming a telescope with a 4:1 de-magnification factor. The vortex beam is centred onto the input facet of a lead-doped glass slab (height *D*=5 mm, length 400 mm and width 40 mm). The near and far fields of the output facet of the glass slab are imaged separately with two lenses onto a camera so as to monitor the real-space intensity distribution and the spatial-frequency spectrum of the simulated boson star. The inset graph shows the heat-induced refractive index change (Δ*n*, normalized to one) in the glass sample as predicted by the full numerical solution to equation (4) (blue dashed line), by the distributed loss model (red line, *σ*=*D*/2) and the experimentally measured Δ*n* (thick black line).

Here *σ* is a nonlocal interaction length scale set by thermal diffusion and can be taken as *σ*∼*D*/2. Indeed, we measured the nonlocal length using the method put forward by Minovich *et al.*^17^, providing evidence that to a good approximation *σ*=*D*/2 in our system, as shown in Fig. 1. Numerical solutions to the thermal diffusion equation indicate that this is a generic feature and not a peculiarity of our system. This type of thermal nonlocality was also previously explored in the context of vortex solitons in ref. 19. With the addition of the distributed loss term, the response function in equation (8) is described by


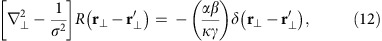


and by Fourier transforming we obtain


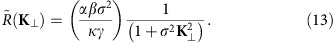


The Fourier transform of equation (6) yields 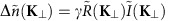, and the effective nonlinear coefficient is given by^20^


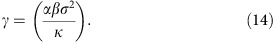


We finally obtain a relation for the K-space response function


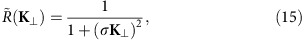


and the corresponding response function in real space


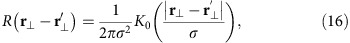


where *K*_0_ is the zeroth-order modified Bessel function of the second kind. In our experiments we use a similar lead-doped glass to that used in ref. 21 with parameters *κ*=0.7 W m^−1^ K^−1^, *n*_b_=1.8, *β*=14 × 10^−6^ K^−1^ and *α*=0.01 cm^−1^. In our experiments described below *D*=5 mm, giving *σ*=2.5 mm and *γ*=1.25 × 10^−6^ cm^2^ W^−1^.

### Comparison of models and reduction to the NSE

We start by noting that when the longitudinal wavevector component *K*_*z*_<<**K**_⊥_, the Coulomb response function in the NSE in K-space reduces to 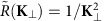. We then recall that for the infinite space model, and using equation (14), the Fourier transform of the response function may be written as 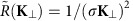, which indeed reproduces the desired Coulomb interaction however, at the expense of a non-physical logarithmic behaviour in real space. The latter is due to the lack of boundaries whose effect is correctly modelled in the distributed loss model (DLM) approach, which provides an exponentially decaying behaviour in direct space. In *K*-space, the distributed loss model response function reduces to the Coulomb form in the limit (*σ***K**_⊥_)^2^>>1. Therefore, for a general input beam (typical laser beams have a Gaussian profile) whose spatial-frequency spectrum includes the region around the origin **K**_⊥_=0, the thermal and the NSE models will be significantly different. However, if we use beams comprising wavevectors only of 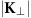
>>1/*σ*, then we may realize the dynamics of the infinite space system, and therefore also of the gravitational system, while still accounting for the boundaries as above. With this caveat, our 2D thermo-optic model has the same response function as the gravitational Poisson equation used in the NSE, thus providing the basis for the correspondence between our nonlocal optical and the Newtonian gravitational system.

Finally, we point out that the propagation along the *z* axis of our optical beam is analogous to propagation in time of a particle in the NSE framework. Furthermore, a gravitational-like potential requires that the sign of Δ*n* is positive.

### Boson stars

We may now use this model to perform experiments that reproduce the dynamics of the NSE. In particular, we chose to study a specific astrophysical object that is described by the NSE, namely a boson star. Originally envisioned by John Wheeler in the 1950s as localized bundles of electromagnetic energy named geons^22^, boson stars are the stable particle-like solution to the Klein–Gordon equation^23^,^24^. Although boson stars may or may not exist in nature they provide a useful testbed for the study of compact objects that can be described by a single wave function. Indeed, boson stars are assumed to be described by a wave function, which is governed by the Einstein–Klein–Gordon equations^16^. The star then arises as a result of a balance between the gravitational field and the dispersion of the wave function. This dispersion essentially originates from the Heisenberg uncertainty principle, that is, the star counteracts gravity due to the impossibility to arbitrarily localize the star's wave function in both position and momentum. In this work we consider a specific type of rotating boson star, which is taken in the weak field (Newtonian) limit where the Einstein–Klein–Gordon equations reduce to the NSE^25^. The fact that a boson star is described by a single-valued wave function implies that the angular momentum must be quantized. More explicitly, the wave function is of the form 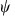
(*r*, *t*, *θ*)=|
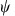
(*r*, *θ*)|*e*^*i*(*ωt*+*mθ*)^, where 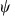
(*r*, *t*, *θ*)=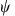
(*r*, *t*, *θ*+2*π*) implies that the angular momentum number *m* is an integer^16^,^26^,^27^,^28^,^29^,^30^,^31^. This in turn leads to a toroidal mass distribution in the star, which can be seen as a result of the fact that the phase is undefined at the star centre^26^,^27^. These features hold in both 3D and 2D (ref. 28) and emerge naturally in optical experiments using beams that carry orbital angular momentum (OAM).

### Experiments

**Figure 2:**
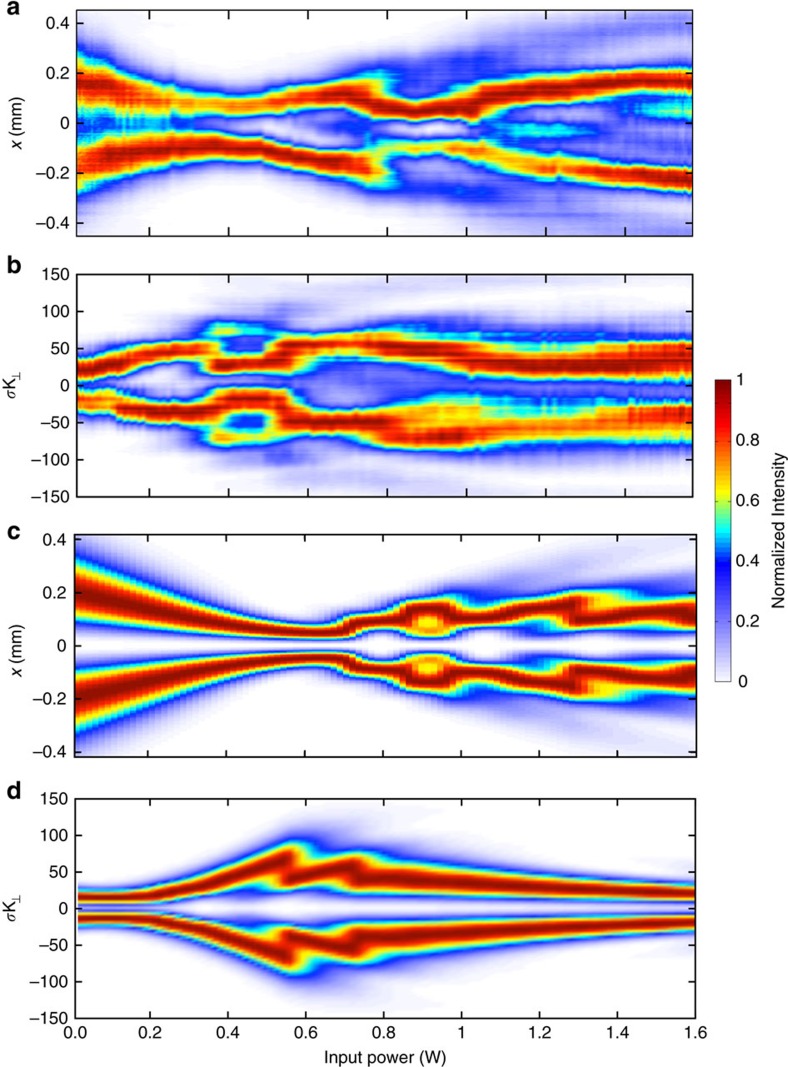
Experiments and numerics studying the dynamics of analogue rotating boson stars. (**a**) The real-space intensity distribution *I*(*x*, 0, *P*) of a vortex beam with 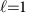 over a propagation distance of 400 mm maps the stable time evolution of a rotating boson star. (**b**) The spatial-frequency spectrum is centred around a value *σ***K**_⊥_∼20, and importantly does not contract to a value smaller than this at any point during the evolution. (**c**,**d**) The numerical simulation of boson star evolution, where **c** is the real-space intensity distribution and **d**, the product of the nonlocal length and transverse **K**_⊥_ vector, *σ***K**_⊥_. While **c** confirms that varying the power reasonably reproduces the evolution as a function of distance, **d** shows that throughout the entire evolution of the vortex beam, *σ***K**_⊥_ remains sufficiently large to reproduce the NSE and therefore simulate a rotating boson star.

A continuous-wave laser with central wavelength 532 nm is used to pump a slab of lead-doped glass with positive thermo-optic nonlinearity (see Methods). We imprint an azimuthal phase onto the Gaussian output beam of the laser via a transmissive phase mask to provide the large wavevectors required to operate in the region (*σ***K**_⊥_)^2^>>1. The beam, now carrying OAM, is imaged after passing through the nonlinear medium onto a charge-coupled device camera (Fig. 1). We monitor both the near and far fields of the output facet of the medium while changing the input power of the beam, *P*. The near field provides the real-space dynamics *I*(**r**_⊥_, *P*) of the boson star while the far field reveals the spatial-frequency spectrum, *I*(**K**_⊥_, *P*). We change the power in the absence of being able to track the beam profile as a function of the propagation distance *z*. At very low beam intensities we see a ring-shaped distribution in both the near and far fields. Figure 2a shows a 2D slice in the *x* plane of the near-field evolution *I*(*x*, 0, *P*) of the vortex beam as the pump power is increased from zero to a maximum power at the sample input facet of ∼1.6 W. The beam starts to self-focus as the power is initially increased, reaching a minimum ring diameter at ∼0.4 W. The ring then expands before collapsing again at ∼0.8 W. It can be seen that there is minimal transfer of energy to higher-order modes suggesting that the energy is predominantly localized within the ring. The far-field evolution is shown in Fig. 2b, which at low intensities has a spectrum centred on a ring with *σ***K**_⊥_≈20. As the far-field spectrum and near-field intensity distribution are inversely linked through their Fourier transform we see that the far field expands at first and contracts after the near field passes through the first minimum at ∼0.4 W. There is some indication of mode splitting hereafter but the majority of the energy is conserved within the single-input mode. We note that throughout the entire propagation the spectrum does not contract to a value smaller than the initial *σ***K**_⊥_, thus ensuring that at all times we are in a regime ((*σ***K**_⊥_)^2^>>1) under which the analogy with the NSE equation is valid. The self-contraction cycles in the optical system are due to the interplay between nonlinearity-induced self-focusing and saturation mechanisms that prohibit the beam to continue collapsing indefinitely. Other optical systems are known to exhibit similar dynamics, for example, laser pulse filamentation, where the saturation and counterbalancing effect is provided by plasma defocusing (see, for example, ref. 32). Here the contraction can be arrested by two mechanisms: the first is diffraction, analogous to the repulsive pressure term due to the uncertainty principle in the gravitational case. This will occur even in the absence of angular momentum. The second is due to the presence of a phase singularity at the centre of the beam (due to the quantized angular momentum) that enforces zero-light intensity there. For gravitational Boson stars, numerical studies have shown similar contraction cycles in the absence of angular momentum^16^,^33^.

### Numerical simulations

We have performed simulations based on equations (5) and (6) using the split-step beam propagation method^34^. In this approach propagation over a length *L* is written as a product of *N*_*z*_ small steps Δ*z*=*L*/*N*_*z*_. For large enough number of longitudinal sample points *N*_*z*_ this allows the propagation to be approximated as a concatenation of small propagation steps of length Δ*z* involving only the diffraction term on the right-hand side of equation (5), followed by the same but involving only the nonlinear term. The small step of diffraction is performed in the transverse spatial-frequency domain using fast-Fourier transforms on a transverse grid of *N*_T_ × *N*_T_ points. The nonlinear refractive index in equation (6) is evaluated by converting the convolution to a product in the spatial-frequency domain and, using equation (15), the resulting small nonlinear step is performed point by point over the transverse plane. For the simulations presented we have used *N*_*z*_=1,000 longitudinal sample points along with *N*_T_==1,024 transverse grid points, convergence being checked by increasing the number of points. We use the same experimental parameters for the material listed above. Thus, for the beam we use an input ring diameter *w*_0_=360 μm, a maximum power input of *P*=1.6 W and 400 mm of propagation distance. Figure 2c,d shows the near-field intensity as a function of the input power. These simulations can then be directly compared with the evolution of optical wavepacket as the power is increased (Fig. 1a,b) with good agreement.

**Figure 3:**
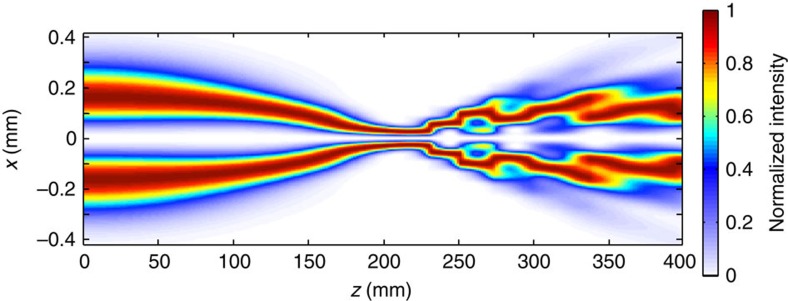
Numerical simulation of experimental boson star evolution in time. We show here the evolution of the optical wave packet according to equation (3). In this case the propagation of the optical field in space (*z*) now maps precisely onto the time coordinate of the boson star evolution.

In Fig. 3 we show the evolution of the boson star in time (that is, intensity distribution along the propagation length of the optical beam *I*(*x*, 0, *z*)). Again, we see an initial contraction of the ring beam followed by a series of oscillations in which the majority of the energy is conserved in the innermost ring, with little lost to higher-order modes.

### Transverse near-field dynamics

**Figure 4:**
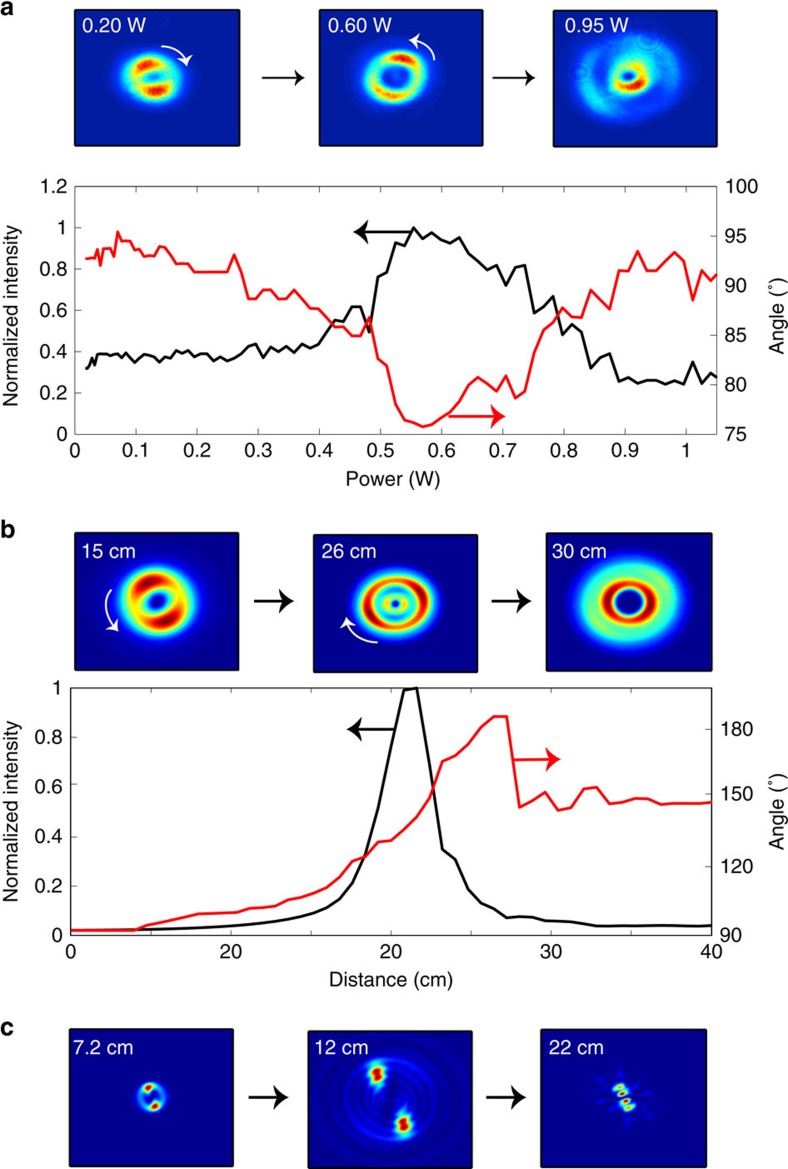
Transverse plane images of an analogue rotating boson star. (**a**) Experiments: the top three panels show examples of the transverse profile of the analogue boson star at different input powers. The graph shows the measured evolution of the peak intensity (left *y* axis, black trace) versus input power for the experiments shown in Fig. 1. The rotation angle of the azimuthal features is shown for increasing input power on the right *y* axis (red trace). (**b**) Numerical simulation of a vortex beam with a similar spatial profile to those seen in the experiments. The rotation angle of the azimuthal features is shown for a fixed input power of 1.6 W for increasing propagation lengths. A similar non-monotonic rotation speed of the peaks is observed as in the experiments. (**c**) Same as in **b**, but for a higher input power of 10 W. The transverse profiles show a more complicated evolution, with a break up into multiple intensity peaks, while the overall coherence is maintained, allowing the contraction to be counteracted by the central phase singularity.

We now consider the transverse profile of the beam. In the experiments we introduce a slight azimuthal anisotropy, which is manifested as two slightly higher-intensity peaks, which live on symmetrically opposite sides of the ring (see top panels in Fig. 4a). Figure 4a shows the maximum intensity in the beam (left *y* axis) and the angle of the peaks as a function of the input power (right *y* axis). The two peaks are seen to slowly rotate around the ring as the beam self-focuses with increasing input power. However, the rotation speed suddenly increases close to the tightest contraction point and then actually inverts direction after this point. This behaviour is confirmed by the numerical simulations shown in Fig. 4b that allow us to observe the same rotation dynamics along the actual propagation axis for a fixed input power of 1.6 W. The non-uniform acceleration of the peaks reveals complicated dynamics for the boson star that has not emerged from previous studies. It is possible to interpret the observed rotation as due to Berry's phase, that is, a geometric phase acquired by the system as it cycles through a closed loop in parameter space. Indeed, based on an analysis of the corresponding linear case (Supplementary Discussion), where we predict a similar rotation effect, we note that the rotation of the peaks appears as a consequence of a Berry phase accumulation that occurs through the beam focus^35^,^36^. Garrison and Chiao^37^ have also shown that geometrical phases will arise in a general class of nonlinear field theories displaying global gauge invariance, as in the case investigated here. Thus, the peak rotation in our experiments and simulations may be viewed as a nonlinear extension of the linear Berry phase occurring during the self-focusing of the fields seen in Fig. 4.

In Fig. 4c we show numerical simulations for a much higher 10 W input power. The boson star is now seen to apparently break up into two well-separated peaks as it contracts. However, these peaks still form a single coherent wave function. Indeed, whilst two independent peaks or boson stars will overlap and create interference fringes^16^, here the merging of these two peaks is prohibited by the input phase singularity, which survives the evolution of the star. Another way of saying this is that the phase singularity acts as a perfectly repulsive point where the momentum of the wave function and thus its kinetic energy diverges to infinity, thus preventing collapse of the star and possibly ultimately limiting the formation of a black hole singularity (Supplementary Discussion).

## Discussion

We have shown that, when appropriate attention is paid to boundary conditions, a medium with positive thermal–optical nonlinearity may provide a testbed for simulations of the NSE. Using optical vortex beams we are able to map the propagation of the wave function along the optical axis to the time evolution of a rotating boson star. However, a thermally focusing medium is not a ‘universal' analogue of the NSE. The nonlocal nonlinearity needs to be properly tailored, for example, by tailoring the input beam shape to enforce the correspondence. We can therefore simulate only a certain subset of objects in the NSE context and we have identified rotating boson stars as one such example that we believe is also of practical interest given that most stars are indeed expected to be in rotation. Therefore, these experiments provide new possibilities for studies into objects described by the NSE. Future work may concentrate on beams with alternate phase profiles, such as Bessel beams and such experiments and studies in the laboratory-based system can provide new insights and research directions for the astrophysical system. Expanding the analysis to include the next higher-order term in the NSE hints that the instabilities observed here will actually be stronger in the full relativistic settings (Supplementary Discussion). It would therefore be interesting to investigate the full relativistic description of phase singularities in rotating objects and their role in the gravitational collapse of boson star-like objects. While our optical analogue can reproduce a 2D slice of the full 3D behaviour of the NSE, an interesting direction would be to look at systems capable of simulating the full 3D NSE. We note that the equations that govern rotating 2D optical beams with a phase singularity at the centre are of the same form as those that describe a rotating boson star, whose geometry is a torus with a similar phase singularity at the centre^16^. As our analogue reproduces a slice of this torus we expect that insight gleaned from our experiments should be transferrable to ‘real-world' cosmological objects.

## Methods

### Experimental details

Our experiments exploring the dynamics of rotating boson stars use a continuous-wave laser with central wavelength 532 nm to pump a slab of lead-doped glass. The Gaussian beam is first passed through an optical fork-patterned phase mask, which imprints an azimuthal phase 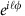, where 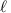 is the diffraction order. In the experiments performed here we use a beam with 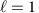, which generates a ring-shaped beam with *I*(**K**_⊥_=0)=0 and large enough transverse wavevectors to be well within the regime (*σ***K**_⊥_)^2^>>1, as required for the correspondence between the NSE and the paraxial wave/heat diffusion equation. We isolate the first 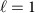 diffracted order with an iris placed in the Fourier plane of a 4:1 telescope, which is also used to reduce the beam input ring diameter to ∼360 μm. The OAM beam is input to a lead-doped glass (Schott SF6) plate with dimensions 5 × 40 × 400 mm (height, width and length); Fig. 1. The output facet of the glass medium is then imaged onto an sCMOS camera (Andor Zyla 4.2+). We simultaneously image both the near- and far-field (spatial-frequency spectrum) intensity profiles of the sample output. The near field is imaged using an *f*_1_=250 mm lens providing a magnification of *M*=2.7. The far field is imaged by placing an *f*_2_=200 mm lens at distance *f*_2_ from both the output surface and from the camera sensor. The transverse wavevector calibration is given by **K**_⊥_=(*x*/*f*_2_)*k*_0_, where *x* is the transverse position and *k*_0_=2*π*/*λ* is the fundamental wavevector. The near and far fields are then monitored as the power of the laser is increased using an attenuator (*λ*/2-waveplate followed by a polarizer) placed before the phase mask.

### Data availability

All experimental and numerical data is available at http://dx.doi.org/10.17861/1b1afd55-46dd-49a1-9ed8-3034bd9a63b1.

## Additional information

**How to cite this article:** Roger, T. *et al.* Optical analogues of the Newton–Schrödinger equation and boson star evolution. *Nat. Commun.* **7,** 13492 doi: 10.1038/ncomms13492 (2016).

**Publisher's note:** Springer Nature remains neutral with regard to jurisdictional claims in published maps and institutional affiliations.

## Supplementary Material

Supplementary Information Supplementary Discussion and Supplementary References
