# Supplementary material for: Optical analogues of the Newton–Schrödinger equation and boson star evolution
Source: Nat Commun. 2016 Nov 14;7:13492. doi: 10.1038/ncomms13492 (PMC5114577; doi:10.1038/ncomms13492)
Supplement: Supplementary Information — Supplementary Discussion and Supplementary References [file ncomms13492-s1.pdf]

Supplementary Discussion

We first expand the concept of Boson star rotation in terms of a geometric Berry phase. We then explore the consequence of including the next-to-leading order general relativistic corrections to the Schrödinger-Newton equation. Finally, we interpret the regular Schrödinger-Newton system as a non-interacting gas coupled to gravity and discuss the behaviour reported in the main text, using this language.

Analysis of the rotating boson stars in terms of a geometric Berry phase: linear case. The rotation of the peaks also occurs to a lesser extent for linear propagation so we look at this first. For a unit OAM strong beam we write the initial condition as ($\epsilon << 1$)

$$\begin{aligned} E(\rho,\theta,z=-L) &= E_0(\rho)e^{i\theta}\left(1+\epsilon\cos(2\theta)\right) \\ &= \underbrace{E_0(\rho)e^{i\theta}}_{\Phi_g(z)} + \frac{\epsilon}{2}\underbrace{E_0(\rho)e^{3i\theta}}_{3\Phi_g(z)} + \frac{\epsilon}{2}\underbrace{E_0(\rho)e^{-i\theta}}_{\Phi_g(z)}. \end{aligned} \tag{1}$$

The three different terms on the lower line have differing OAM $\ell = +1, 3, -1$, and therefore accumulate differing Gouy phase-shifts $|\ell|\Phi_g(z)$ through the focus, with $\Phi_g(z)$ the Gaussian beam Gouy phase. It is the z-dependent phase differential between the $\ell = 3, -1$ terms that allows the peaks to rotate. To see this qualitatively, if one ignores the radial field dependence for simplicity ($\epsilon << 1$)

$$|E(\theta,z)|^2 \simeq 1 + 2\epsilon\cos(\Phi_g(z))\cos(2\theta + \Phi_g(z)). \tag{2}$$

Now $-\frac{\pi}{2} < \Phi_g(z) < \frac{\pi}{2}$, so $\cos(\Phi_g(z)) > 0$, and the peaks follow $\theta_p(z) = -\frac{\Phi_g(z)}{2}$ which changes sign through the origin. (Note that due to the neglect of radial modes this is approximate at best.)

So in the linear case the Gouy phase-shift is at the heart of the rotation of the peaks. On the other hand it is known that the Gouy phase is a manifestation of a general Berry phase that occurs through a beam focus when the beam parameters undergoe cyclic motion [1, 2]. The Berry phase is a geometric or topological phase acquired by a system after if is cycled though a closed loop in parameter space. So the rotation during focusing may be interpreted as a geometric phase effect in the linear case.

Analysis of the rotating boson stars in terms of a geometric Berry phase: nonlinear case. The rotation of the peaks survives in the nonlinear case and appears modified but enhanced due to the nonlinear self-focusing effect. We cannot offer a detailed analysis of this but we can point to some general expectations that support that the rotation remains a Berry phase effect. In his paper Subbarao [1] already mentioned that the Berry phase would persist in the Gaussian beam analysis of self-focusing. Furthermore, Garrison and Chiao [3] argued that geometrical phases will arise in a general class of nonlinear field theories displaying global gauge invariance, and this applies in our case. Furthermore their work is not restricted to the adiabatic approximation, so the notion of Berry's phases resulting from nonlinear self-focusing is expected to persist. Thus the peak rotation in the nonlinear case may be viewed as a nonlinear extension of the linear case whose physical origin lies in the nonlinear Berry's phase during the self-focusing of the fields.

Effects of General Relativity to the Schrödinger-Newton system. We wish to show that the next-to-lowest order corrections to the NSE, indicate that the full general relativistic description will lead to the similar results as those shown in this work, albeit with a stronger attractive forces. Let us start with deriving the Schrödinger-Newton equation from the Klein-Gordon coupled to the weak-field limit of general relativity. In the latter mentioned limit, one can show that Einstein's field equations reduce to

$$\nabla^2\Phi = 4\pi G m \Psi\Psi^* \tag{3}$$

where the metric tensor components are given by

$$g_{00} = -(1+2\Phi), \quad g_{ij} = (1-2\Phi)\delta_{ij}, \quad i,j = 1,2,3. \tag{4}$$

The coupling to the Klein-Gordon equation then follows:

$$\hbar^2\left[-(1-2\Phi)\partial_t^2 + c^2(1+2\Phi)\nabla^2\right]\Psi - m^2c^4\Phi = 0. \tag{5}$$

Here we assume that $\Phi \ll 1$ and that Φ^2 is negligible. We can now proceed with the ansatz $\Psi = \psi e^{-i\frac{mc^2}{\hbar}t}$ in eq. 5. By neglecting the $\mathcal{O}\left(c^{-2}\right)$ term (i.e. slow-moving limit) we arrive at

$$i\hbar\left[1-2\Phi\right]\partial_t\psi = -\left[1+2\Phi\right]\frac{\hbar^2}{2m}\nabla^2\psi + mc^2\Phi\psi \tag{6}$$

This is the Schrödinger-Newton equation but where the next-to-leading order effects of gravity are taken into account. We can see that by neglecting $\Phi\psi$ products, we get the regular Schrödinger-Newton equation. The main difference wth respect to the NSE is that general relativity acts to 'amplify' the momentum, that is, $k^2 \to k^2(1+\Phi)$ when next-to-leading order effects are taken into account. Unstable solutions of the Schrödinger-Newton equation are thus unstable also when general relativistic effects are taken into account.

Schrödinger-Newton as a non-interacting gas. We start from the regular Schrödinger equation with a potential U.

$$i\hbar\partial_t\psi = -\frac{\hbar^2}{2m}\nabla^2\psi + U\psi \tag{7}$$

It is well know that by a Madelung transform, i.e. $\psi = \sqrt{\rho}e^{iS}$, we can recast this into hydrodynamical equations describing a non-interacting gas:

$$\partial_t\rho + \boldsymbol{\nabla}\cdot(\rho\vec{v}) = 0 \tag{8}$$

describing probability conservation, and

$$\partial_t v + \boldsymbol{\nabla}\left[\frac{1}{2}v^2 + \frac{U}{m} - \frac{\hbar^2}{2m^2}\frac{\nabla^2\sqrt{\rho}}{\sqrt{\rho}}\right] = 0 \tag{9}$$

describes the 'velocity' profile. Note that the 'velocity' of the gas is here given by $\mathbf{v} = \frac{\hbar}{m}\boldsymbol{\nabla}S$. First we can see that there is an outward pressure, usually denoted quantum pressure, given by $P_Q = \frac{\hbar^2}{2m^2}\frac{\nabla^2\sqrt{\rho}}{\sqrt{\rho}}$, which is relevant when the density grows rapidly.

Now we let this 'gas' gravitate. Or in other words, we let $U = mc^2\Phi$, where Φ is determined by eq. 3. Substituting this into eq. (9), we find

$$\partial_t v + \boldsymbol{\nabla}\left[\frac{1}{2}v^2 + c^2\Phi - \frac{\hbar^2}{2m^2}\frac{\nabla^2\sqrt{\rho}}{\sqrt{\rho}}\right] = 0 \tag{10}$$

which is coupled to

$$\nabla^2\Phi = 4\pi G m\rho. \tag{11}$$

We note that in the present context ρ is *not* a mass density, but a probability density.

Conclusions drawn from the gas picture. The above equations are very similar to the hydrodynamical equations commonly used when discussing star formation. However, there are some important differences. The gas we see above is non-interacting. This implies that there is no direct pressure arising from 'particle' bunching, which is required for normal hydrostatic equilibrium. Hydrostatic equilibrium is however still possible, but gravity now needs to be balanced by the quantum pressure P_Q. Boson stars are indeed stable due to the Heisenberg uncertainly principle [4], and here we see that this manifests itself naturally through the quantum pressure term in the hydrodynamical picture.

We now focus attention on the implications of the fact that for a wavefunction with angular momentum, there is a phase singularity at the centre. At this central point, the hydrodynamical picture is not well defined. However, we can see the effective probability 'gas' will naturally avoid this point, as there the term $\frac{1}{2}v^2 \to \infty$ in eq. (10). The centre is thus a point of infinite repulsive pressure, which is just another description of a perfect mirror. Another viewpoint is that this point has infinite potential energy, as we see that it enters in the same way as the gravitational potential Φ in eq. (10). Either way, our conclusions remain the same: any 'particles' that encounter the centre will bounce away from it.

Supplementary References

[1] Subbarao, D. Topological phase in Gaussian beam optics. Opt. Lett. **20**, 2162 (1995).

[2] Feng, S. & Winful, H. G. Physical origin of the Gouy phase-shift. Opt. Lett. **26**, 285 (2001).

[3] Garrison J. C. & Chiao, R. Y. Geometrical phases from global gauge invariance of nonlinear classical filed theories. Phys. Rev. Lett. **60**, 165 (1988)

[4] Liebling, S.L. & Palenzuela, C. Dynamical boson stars, Living Rev. Relativity, **15**, 6 (2012)
